# A multi-omics-based investigation of the prognostic and immunological impact of necroptosis-related mRNA in patients with cervical squamous carcinoma and adenocarcinoma

**DOI:** 10.1038/s41598-022-20566-0

**Published:** 2022-10-06

**Authors:** Jiani Zou, Zhiheng Lin, Wenjian Jiao, Jun Chen, Lidong Lin, Fang Zhang, Xiaodan Zhang, Junde Zhao

**Affiliations:** 1 Shandong University of Traditional Chinese Medicine, Jinan, 250014 Shandong China; 2 Department of Traditional Chinese Medicine, Qilu Hospital of Shandong University, Jinan, 250012 China

**Keywords:** Cancer, Immunology, Molecular biology, Biomarkers, Oncology

## Abstract

Necroptosis is a kind of programmed necrosis mode that plays a double-edged role in tumor progression. However, the role of necroptosis-related Messenger RNA (mRNA) in predicting the prognosis and immune response of cervical squamous carcinoma and adenocarcinoma (CESC) has not been fully studied. Firstly, the incidence of somatic mutation rate and copy number variation for 74 necroptosis-related mRNAs (NRmRNAs) were analyzed. Secondly, CESC patients were divided into four stable clusters based on the consensus clustering results and analyzed for correlations with a series of clinical factors. Subsequently, a total of 291 The Cancer Genome Atlas samples were randomly divided into either training or validation cohorts. A Cox proportional hazard model consisting of three NRmRNAs (CXCL8, CLEC9A, and TAB2) was constructed by univariate, least absolute shrinkage and selection operator and multivariate COX regression analysis to identify the prognosis and immune response. Its performance and stability were further validated in another testing dataset (GSE44001) from Gene Expression Omnibus database. The results of the receiver operating characteristic curve, principal component analysis, t-SNE, and nomogram indicated that the prognostic model we constructed can serve as an independent prognostic factor. The combination of the prognostic model and the classic TNM staging system could improve the performance in predicting the survival of CESC patients. In addition, differentially expressed genes from high and low-risk patients are screened by R software for functional analysis and pathway enrichment analysis. Besides, single-sample gene set enrichment analysis revealed that tumor-killing immune cells were reduced in the high-risk group. Moreover, patients in the low-risk group are more likely to benefit from immune checkpoint inhibitors. The analysis of tumor immune dysfunction and exclusion scores, M6A-related genes, stem cell correlation and Tumor mutational burden data with clinical information has quantified the expression levels of NRmRNAs between the two risk subgroups. According to tumor immune microenvironment scores, Spearman’s correlation analysis, and drug sensitivity, immunotherapy may have a higher response rate and better efficacy in patients of the low-risk subgroup. In conclusion, we have reported the clinical significance of NRmRNAs for the prognosis and immune response in CESC patients for the first time. Screening of accurate and effective prognostic markers is important for designing a multi-combined targeted therapeutic strategy and the development of individualized precision medicine.

## Introduction

Since the beginning of the twenty-first century, cervical cancer (CC) has become a malignant tumor that seriously endangers the health of women all over the world. Its incidence has been increasing year by year^1^. According to the International Agency for Research on Cancer (IARC), the number of new cases of cervical cancer worldwide was 604,127 in 2020, accounting for 6.5% of newly developed female malignant tumors worldwide (ranked fourth). The death tolls were more than 300,000^2^. Of more than 80% of new cases and deaths occurred in underdeveloped countries and regions^3^ with backward economic, health and medical conditions. CESC is the most common histological subtype of the CC, accounting for approximately 15%^4^ of deaths due to tumors in women. Although the incidence of cervical cancer has been greatly reduced because of cancer screening^5^ and HPV vaccination^6^ in the past few decades, the clinical indicators used to determine the prognosis are still imperfect. The mortality rate of patients with advanced CESC is still not optimistic. Therefore, identifying more biomarkers that are able to accurately evaluate the condition of CESC is of great significance for the clinical development of a personalized and accurate diagnosis and treatment plan and the improvement of prognosis.

Necroptosis, a programmed necrosis pattern^7^ highly dependent on the regulation of intracellular signaling pathway, is characterized by rapid loss of plasma membrane integrity and release of the proinflammatory cell contents^8^, as well as releasing damage-associated molecular patterns and stimulating immune response^9^. Receptor Interacting Serine/Threonine protein [RIP] Kinase 1 (RIPK1)^10^ and RIPK3^11^ play an important regulatory role in the signaling cascade of necroptosis. The RIPK1/RIPK3 complex recruits and phosphorylates the mixed-lineage kinase domain-like protein (MLKL), thereby triggering necroptosis of cells. Several studies have discovered that the three key proteins (RIPK1, RIPK3 and MLKL) in the necroptosis signaling pathway are expressed in a variety of tumor cell lines^12^ and play an important role^13^ in the progression of tumor. As cells undergo necroptosis, it may eventually lead to cell membrane rupture and release a series of damage-associated molecular patterns (DAMPs). Necroptosis is also considered as a pro-inflammatory cell death^14^. In the inflammation induced by DAMPs, several signaling pathways such as NF-B or MAPK pathways are activated. Some studies suggest that their activation may play a tumor-promoting role^15,16^. For example, RIPK3-mediated necroptosis promotes the chronic inflammation and the occurrence in colorectal tumors^17^. On the other hand, the release of DAMPs after cells undergo necroptosis also promotes DC maturation and cross-presentation of CD8^+^ T cells in the TIME, which subsequently induce the anti-tumor immunity^13,18^. For example, upregulation of MLKL expression in cervical squamous carcinoma predicts a low histological grade, the reduction of metastatic spread and the improvement of overall survival^19^. Therefore, increasing evidence indicates that the necroptosis plays a double-edged sword role in tumor progression, either anti-tumor or promoting tumor development. However, the specific role and mechanism of the necroptosis remain unclear in the occurrence and progression of CESC and metastasis. It is necessary to screen and identify more novel biomarkers related to necroptosis, so as to provide more references and options for the treatment of CESC.

Increasing evidence of frontier tumor molecular mechanisms suggests that RNA plays a crucial role^20^ in many cell biological processes that affect the associated tumorigenesis and malignant progression. However, the role of necroptosis-related mRNA in predicting the prognosis of CESC has not been fully studied. In this study, we have first established four cluster subtypes and systematically analyzed the relationship with mutations, CNVs, TME, prognosis and immunity based on the sequencing data of NRmRNAs from CESC samples. In the next place, we have adopted TCGA to construct the first 3-NRmRNAs risk score model with independent prognostic value and deeply evaluated the clinical significance of this prognostic model so as to provide potential biomarkers for the diagnosis and prognosis of CESC, thus helping guiding personalized precision treatment clinically.

## Materials and methods

### Visualization of mutations and CNV

Mutational data such as somatic mutations and CNV data were downloaded from UCSC Xena (https://xena.ucsc.edu/). Mutation annotation format (maf) data were processed and analyzed using the MutSigCV algorithm and the "maftools" software package. A waterfall plot was used to visualize the mutational information of NRmRNAs from CESC patients in the TCGA database. The CNV frequency figures were visualized using the "barplot" command in the R language, with the abscissa as the name of NRmRNAs and the ordinate as the frequency of the CNV corresponding to NRmRNAs. Circles of CNV frequencies were drawn using the "RCircos" package (Red: high frequency of increased NRmRNAs copy number; Blue: high frequency of deleted NRmRNAs copy number).

### Consensus clustering analysis

The most variable gene in CESC cells in response to necroptosis-related gene sets in TCGA cohort was determined according to the median absolute deviation (MAD). Consensus clustering analysis of CESC samples was performed using both "limma" and "consensusClusterPlus" packages based on uclidean distance and Wards linkage. In unsupervised analysis, the quantitative stability evidence was obtained based on cumulative distribution function (CDF) in order to further confirm the optimal number of clusters. Survival analysis was carried out for CESC patients upon samples data using the "survival" and "survminer" function packages, where the abscissa represented the survival time and the ordinate represented the survival rate.

### Tumor microenvironment based on immune and matrix scores

In order to analyze the expression levels of immune-cell and stromal-cell-specific genes in the tumor microenvironment (TME), the "ESTIMATE" algorithm was used to calculate the matrix scores and immune scores for these 4 types of samples. The scores were obtained based on the gene expression of CESC patients in the TCGA database and can be downloaded through the online website (https://bioinformatics.mdanderson.org/estimate/disease). The infiltration of stromal cells and immune cells in TME was inferred by analyzing transcriptomic data from tumor samples. The data visualization was completed using "reshape2" and "ggpubr" program packages.

### Correlation and distribution difference analysis between NRmRNAs and immune-infiltrating cells of CESC

CIBERSORT is widely used to assess the type of immune cells in the microenvironment. This tool is able to perform a deconvolution analysis for the expression matrix of immune cell subtypes based on the principle of linear support vector regression. It contains 547 biomarkers and also defines 22 human immune cell phenotypes covering plasma, B cells, T cells and myeloid cell subsets. Immune cell gene expression profiles and the CIBERSORT calculation R package were downloaded from the CIBERSORT website to quantify the immune cell infiltration. Heatmaps and pairwise difference plots were drawn for the immune cell infiltration data using the R package.

### Construction and validation of the prognostic model of necroptosis-related mRNA

A total of 291 TCGA samples were randomly divided into two groups: train group (n = 147) and test group (n = 144). In order to identify the prognostic value of NRmRNAs, the NRmRNAs significantly related to the OS in patients with CESC were screened for the samples in the train group by univariate Cox regression analysis (*P* < 0.05). In order to avoid over-fitting with the range of precise prognostic NRmRNAs candidates, we performed the Cox regression analysis of LASSO using the "glmnet" of R software, as well as tenfold cross-validation. Next step, the NRmRNAs with prognostic value were screened based on the results of the multivariate Cox regression analysis. Meanwhile, the regression coefficients derived from the regression model were output. A risk score formula was established based on the expression level of each NRmRNAs and its corresponding regression coefficient, constructing a risk score model for predicting CESC patients. Hazard scores of the prognostic risk score model (X: coefficient, Y: gene expression level) = $\sum_{\mathrm{i}}^{\mathrm{n}}\mathrm{Xi}\times \mathrm{Yi}$

### Construction and performance evaluation of the prognostic risk model

CESC patients were classified into high and low risk groups based on the median risk score. The OS of the two risk subgroups was analyzed and compared using the "survival" package. Moreover, the dual elements, time and clinical ROC curves, were further analyzed for this risk score model using the "time ROC" and "Clinical ROC" packages so as to evaluate the predictive accuracy of this model. The mRNA expression profiles and complete overall survival (OS) data of another 300 tumor patients (GSE44001) were obtained from the GEO database (https://www.ncbi.nlm.nih.gov/geo/query/acc.cgi) as independent validation set. PCA and t-SNE analysis was performed using the prcomp function to discuss the data distribution of the two hazard subgroups. The results of the two subgroups were visualized using the "rtsne" and "ggplot2" software packages.

Univariate and multivariate Cox regression analyses were performed to assess the correlation between the variables (such as physiologic factors, pathological stages and risk scores) and the prognosis and to identify whether this risk model could be used as an independent prognostic indicator for CESC patients. The rms, survival and so on software packages of R software were used to establish and create a nomogram to predict the prognosis of CESC patients and draw a correction curve, so as to compare and validate the 1-year, 3-year and 5-year survival rates in the nomogram.

### Risk score model versus classic TNM staging system

Evaluate the correlation between classic TNM staging system and risk score. Stratified OS analyses were conducted to assess the differences in the subgroups of early clinicopathology classifications including T1 stage, T2 stage, T3 stage, T4 stage, TX stage, N0 stage, N1 stage, NX stage, M0 stage, M1 stage and MX stage.

### Functional enrichment analysis

In order to investigate the mechanisms of occurrence and development of CESC, the gene function analysis (gene ontology, GO) and Kyoto Encyclopedia of Genes and Genomes (KEGG) pathway analysis were introduced to annotate and describe the function of gene products in detail. GO covers molecular biological functions (MF), cytological components (CC) and biological processes (BP). The functional information of a specified gene is comprehensively summarized in the form of enrichment analysis. KEGG is a database that integrates genomic, chemical and systemic functional information and systematically analyzes the gene function in terms of gene and molecular networks. It is commonly used to identify functional and metabolic pathways. The annotation of differentially expressed genes in GO was analyzed using the 'clusterProfiler' package and the pathway analysis was carried out via KEGG.

### Gene enrichment analysis

In the interest of the elucidation of relevant pathways and biological processes in the NRmRNAs high and low risk groups, the gene set enrichment analysis (GSEA) (version 4.1.2) software was used to analyze the expression gene sets of low-risk and high-risk groups and the marker gene sets collected from the KEGG database V7.5. fdr < 0.05 was defined as statistical significance.

### Immunological correlation analysis of NRmRNAs

ssGSEA analysis was performed using the "GSVA" R package to calculate the infiltration scores for 16 immune cells and 13 immune pathways in CESC. Then, the activity of immune checkpoints was investigated in the high and low risk groups. In order to explore the relationship between the m6A RNA methylation regulators and CESC between the two risk subgroups, boxplots were drawn using the "ggboxplot" commands. The prognostic effect of immune checkpoint inhibitory therapy was further evaluated by TID scores. The "ggExtra" and "ggpubr" of R package were applied to calculate the correlation of stem cells of the two-risk subgroups. The TIME scores (including matrix and immune scores) of samples were calculated for CESC patients using the "ESTIMATE" package. The data results were visualized by "reshape2" and "ggpubr" packages. For further investigation of the correlation between the immune cell, stromal cell scores and the risk scores, we performed Spearman analysis for the two sets of data.

### Grouping and survival analysis of tumor mutation burden

Genetic mutation maps of CESC patients were drawn using the “maftools” R package in two risk subgroups. *Perl* software was used to determine the total number of gene mutations in each sample in order to calculate the TMB. Genetic mutations included coding shifts, base substitutions, deletions and insertions. The difference in TMB was compared between the high and low risk groups. The correlation was analyzed between the TMB levels and risk scores. HCC samples were divided into high TMB group (n = 136) and low TMB group (n = 136) based on the best cutoff generated by *X-Tile* software (version 3.6.1). The relationship of overall survival was analyzed by Kaplan–Meier for CESC patients among the TMB subgroup, the high and low risk groups.

### Drug sensitivity of two-risk subgroups

"Limma" and "pRRophetic", these two R packages were applied to analyze the drug sensitivity data of two risk subgroups and the results were visualized using "ggpubr", "ggplot2" and "ggboxplot". Where the abscissa represented the risk value of CESC patients (Blue: low risk group; Red: high risk group) while the ordinate represented the drug sensitivity.

## Results

**Figure 1:**
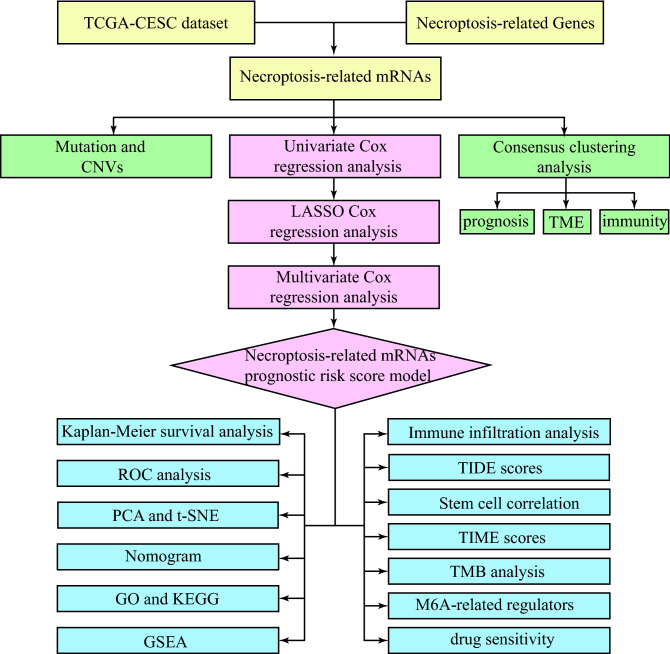
Flow diagram of full-text data.

The research flow of this paper is shown in Fig. 1. Firstly, we have downloaded RNA sequencing, somatic mutations, and CNV data of CESC patients from TCGA. Secondly, consensus clustering analysis is performed based on the response of CESC cells to necroptosis-related gene sets. Once again, a total of 291 TCGA samples are randomly divided into either training or validation cohorts. In the training cohort, a Cox proportional hazard model consisting of three NRmRNAs (CXCL8, CLEC9A, and TAB2) is constructed by univariate, LASSO, and multivariate COX regression analysis to identify the prognosis and immune response. Its prognostic value is further measured by Kaplan–Meier survival analysis, ROC curve, PCA, t-SNE, and nomogram in the validation cohort. Its performance and stability were further validated in another testing dataset (GSE44001) from Gene Expression Omnibus (GEO) database. DEGs from high and low-risk patients are screened by R software for functional analysis and pathway enrichment analysis. The combination of CIBERSORT, ssGSEA, TIDEscores, and stem cell correlation has quantified the TIME scores, the infiltration level of immune cells, the immune checkpoint activity, and the immune-related function. The TMB level is calculated for both risk subgroups and analyzed for their relationship with OS. In addition, we have evaluated the m6A-related genes and the correlation between the drug sensitivity and risk scores.

### Genetics and variation of CESC

**Figure 2:**
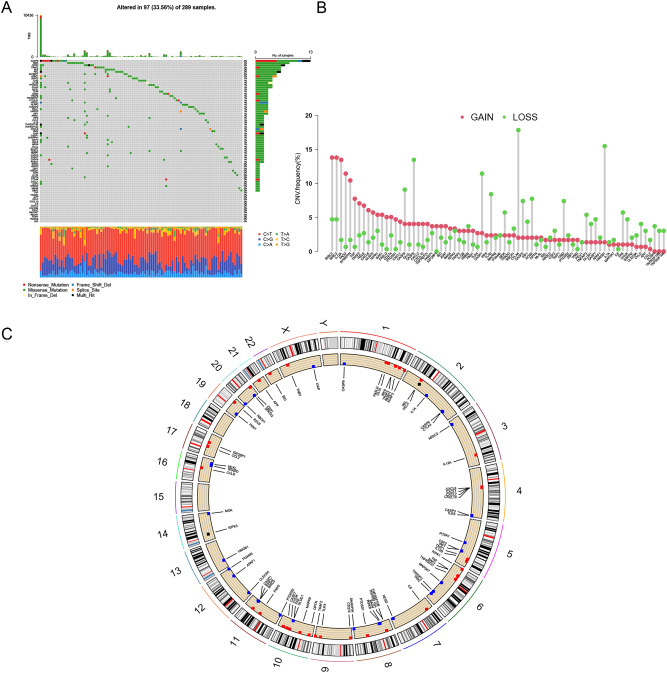
Mutations and CNV information of 74 NRmRNAs in CESC. (**A**) Ninety-seven out of 289 patients show different genetic alterations, including nonsense mutations, splice-site mutations and missense mutations. (**B**) CNV of 74 NRmRNAs: Columns: CNV frequency; Red dots: CNV amplification; Green dots: deletion of CNV. (**C**) Location of CNV alterations in the intracellular NRmRNAs: Red: high frequency of increased NRmRNAs copy number; Blue: high frequency of deleted NRmRNAs copy number.

The waterfall plot shows the somatic mutation rate and the incidence of copy number variation for 74 NRmRNAs. As shown in Fig. 2A, 97 (33.56%) of 289 CESC patients had mutations, among which nonsense mutations, splice site mutations and missense mutations were the main mutations. Moreover, CASP8 mutations had the highest frequency, followed by TAB3, BIRC2, APAF1, IGF2BP1, REL, FAS, TRAF2, NOS3. Whereas SPATA2, APP, TRADD, TNF, BAK1 and IKBKB did not show any mutations. We further investigated the frequency of CNV occurrence in the 74 NRmRNAs totally. As shown in Fig. 2B, the incidence of copy number variation in NRmRNAs was generally increased with a small number of deletions, of which IL12A, FADD, SHARPIN, BIRC3, and BIRC2 had significant CNV amplification frequencies. Figure 2C showed the visualized CNV expression of all NRmRNAs in the CESC samples.

### Genotyping based on the response of CESC cells to necroptosis-related gene sets

**Figure 3:**
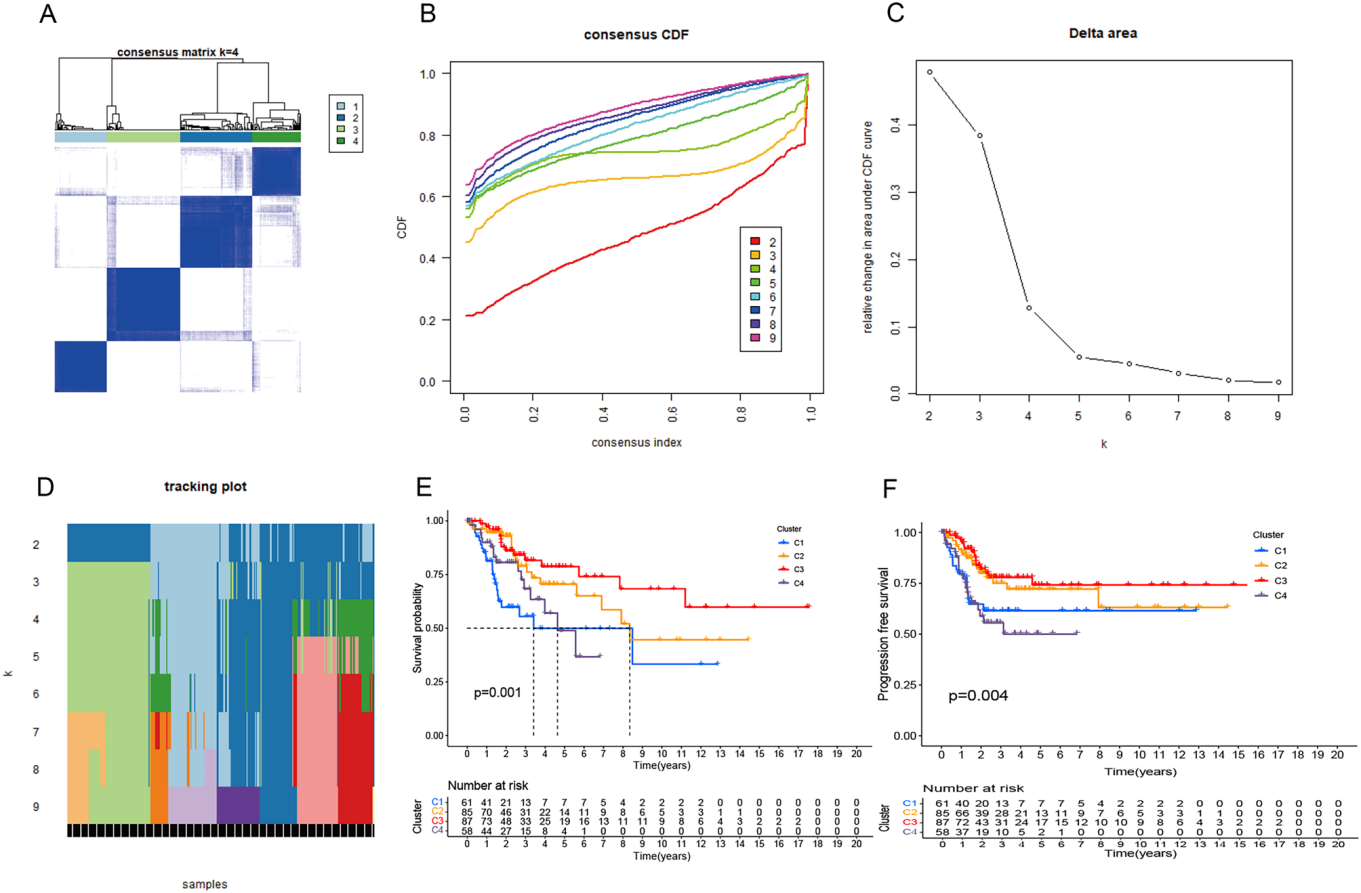
Genotyping of CESC cells based on their response to necroptosis-related gene sets. (**A**) Consensus clustering matrix of the 291 CESC samples with k = 4. (**B**) Consensus cluster CDF from k = 2 to k = 9. (**C**–**D**) Relative change in the area under curve CDF from k = 2 to k = 9. (**E**–**F**) Kaplan–Meier method survival analysis of four clusters.

The consensus clustering results in Fig. 3A indicated that the 291 patients in the TCGA cohort could be divided into four stable clusters. In addition, the deep internal color of four kinds of typing in this distance nomogram represents high correlation while the light color between subtypes represents low correlation. In Fig. 3B, the abscissa represents the number of groups while the ordinate represents the cumulative distribution function (CDF). The greater the CDF value is, the higher the accuracy will be. There is a significant difference in CDF added value when the ordinate is at 4 in the figure, which further validates the rationality of the typing. Cluster stability increased between k = 2 and k = 9 (Fig. 3B–D). The Kaplan–Meier survival analysis showed decreased survival in CESC patients over time. A *P* value less than 0.01 indicated that the patient survival varied between the subtypes. As shown in the figure, the typed Cluster 3 (C3) samples had the longest survival time (Fig. 3E), the longest progression-free survival (PFS) and the best prognosis (Fig. 3F).

### Analysis of tumor microenvironment and immune cell infiltration

**Figure 4:**
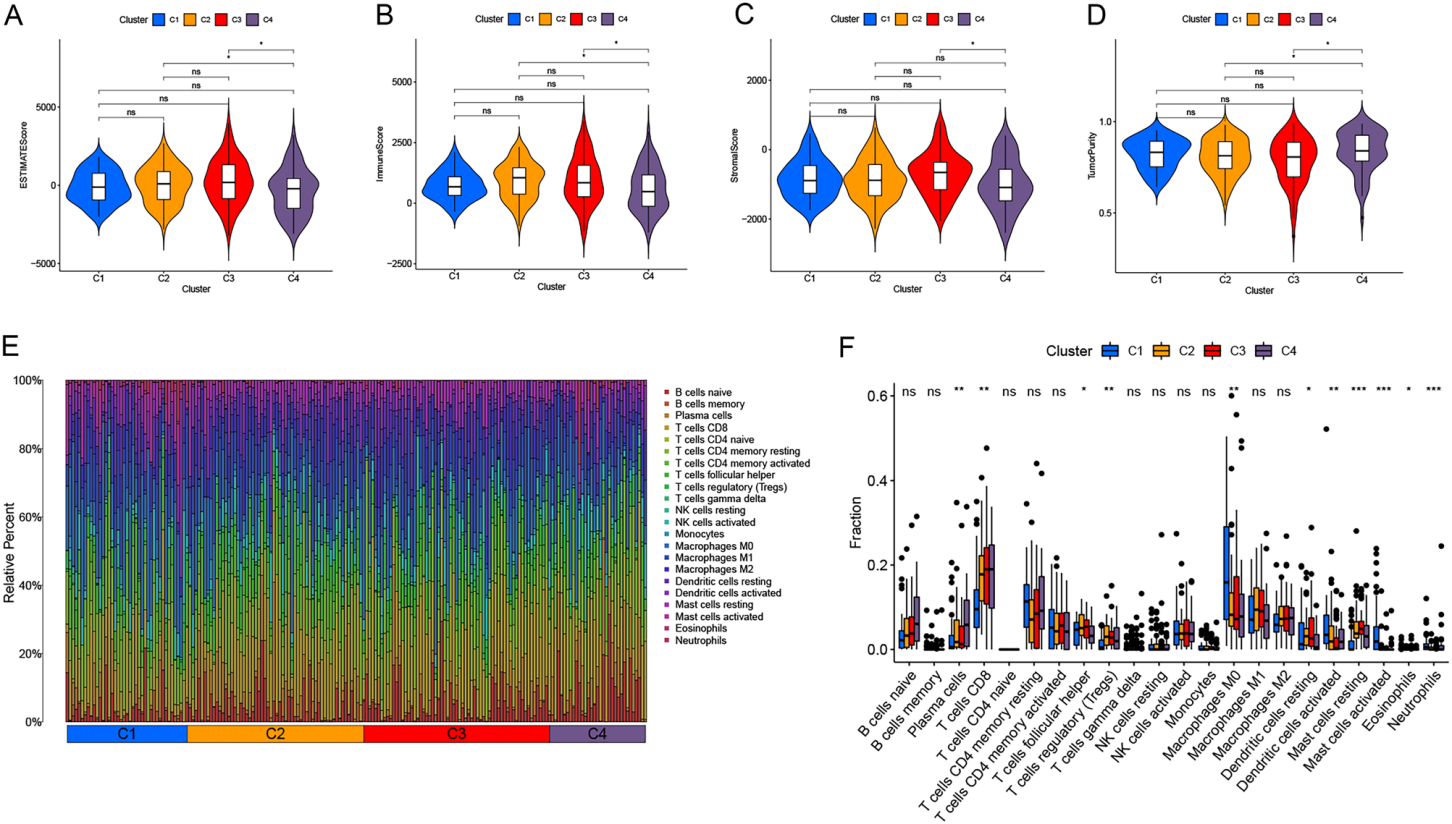
Correlation of immune scores and matrix scores and analysis of immune cell infiltration. (**A**) ESTIMATEScore; (**B**) ImmuneScore; (**C**) StromalScore; (**D**) TumorPurity; (**E**) Heat maps of principal component analysis for the immune cell infiltration among the four types; (**F**) Differential analysis of immune cell infiltration distribution among the four types.

As shown in Fig. 4A–B, ESTIMATEScore and ImmuneScore were significantly correlated with types C2-4 (all *P* < 0.05). StromalScore was significantly correlated with types C3-4 (*P* < 0.05) (Fig. 4C). TumorPurity was significantly correlated with types C2-4 (*P* < 0.05) (Fig. 4D). Immune cell infiltration matrix was obtained via CIBERSORT deconvolution algorithm for the gene expression profile of samples. Principal component analysis was carried out for the immune cell infiltration matrix of four types. Figure 4E showed there was a significant difference in the immune cell infiltration among the four types. Of M0 macrophages had the highest proportion in CI; T cells regulatory (Tregs) had the highest proportion in C2; NK cells activated had the highest proportion in C3; and T cells CD8 had the highest proportion in C4. Pairwise differential analysis of the same immune cell infiltration of the four types suggested that plasma cells, T cells CD8, dendritic cells activated, T cells regulatory (Tregs) and macrophages M0 were significantly different among the four types (all *P* < 0.01). See Fig. 4F.

### Screening and validation of prognostic NRmRNAs

**Figure 5:**
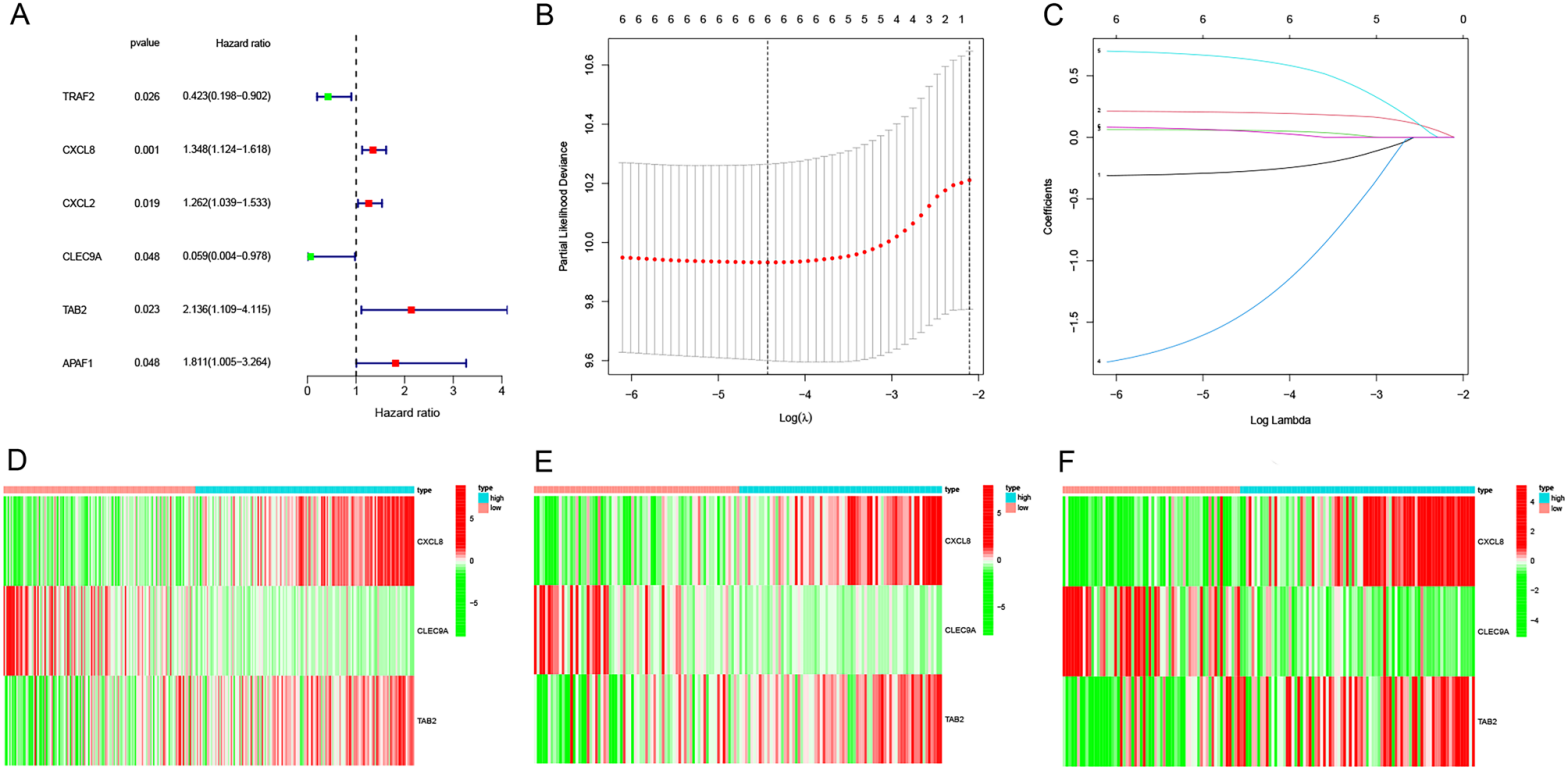
Screening for prognosis-related NRmRNAs in CESC patients. (**A**) Univariate COX regression analysis. (**B**–**C**) Lasso Cox regression analysis. (**D**-**F**) Heatmaps with two sets of validation datasets of the 3 NRmRNAs (green: low expression level; red: high expression level).

A total of 291 CESC patients from the TCGA database were finally included in this study. Based on previous reviews and related research literature, the expression data of 74 NRmRNAs were extracted and the univariate COX regression analysis was conducted for the OS. Six NRmRNAs significantly correlated with overall survival were initially selected (*P* < 0.0001) (Fig. 5A), resulting in six prognosis-related NRmRNAs for LASSO COX regression analysis to establish the prognostic model. A prognostic model with 6 genes was determined based on the optimal λ value (Fig. 5B–C). Further multivariate Cox regression analysis was used to ultimately obtain a prognostic model consisting of three NRmRNAs (CXCL8, CLEC9A and TAB2) (Fig. 5D–F).

### Construction of the prognostic model

**Figure 6:**
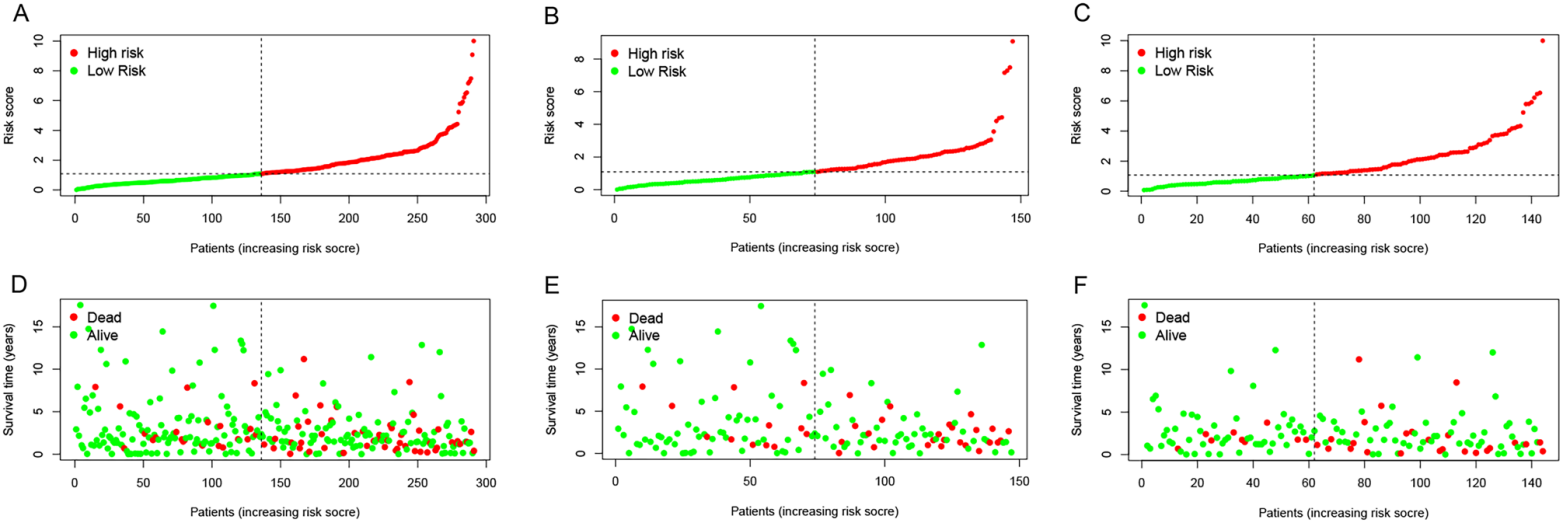
Distribution of risk scores in CESC patients. (**A**–**C**) Distribution and median value of risk scores of patients in the TCGA cohort and two validation data sets; (**D**–**F**) Distribution of OS status and risk scores of patients in the TCGA cohort and the two validation data sets.

**Figure 7:**
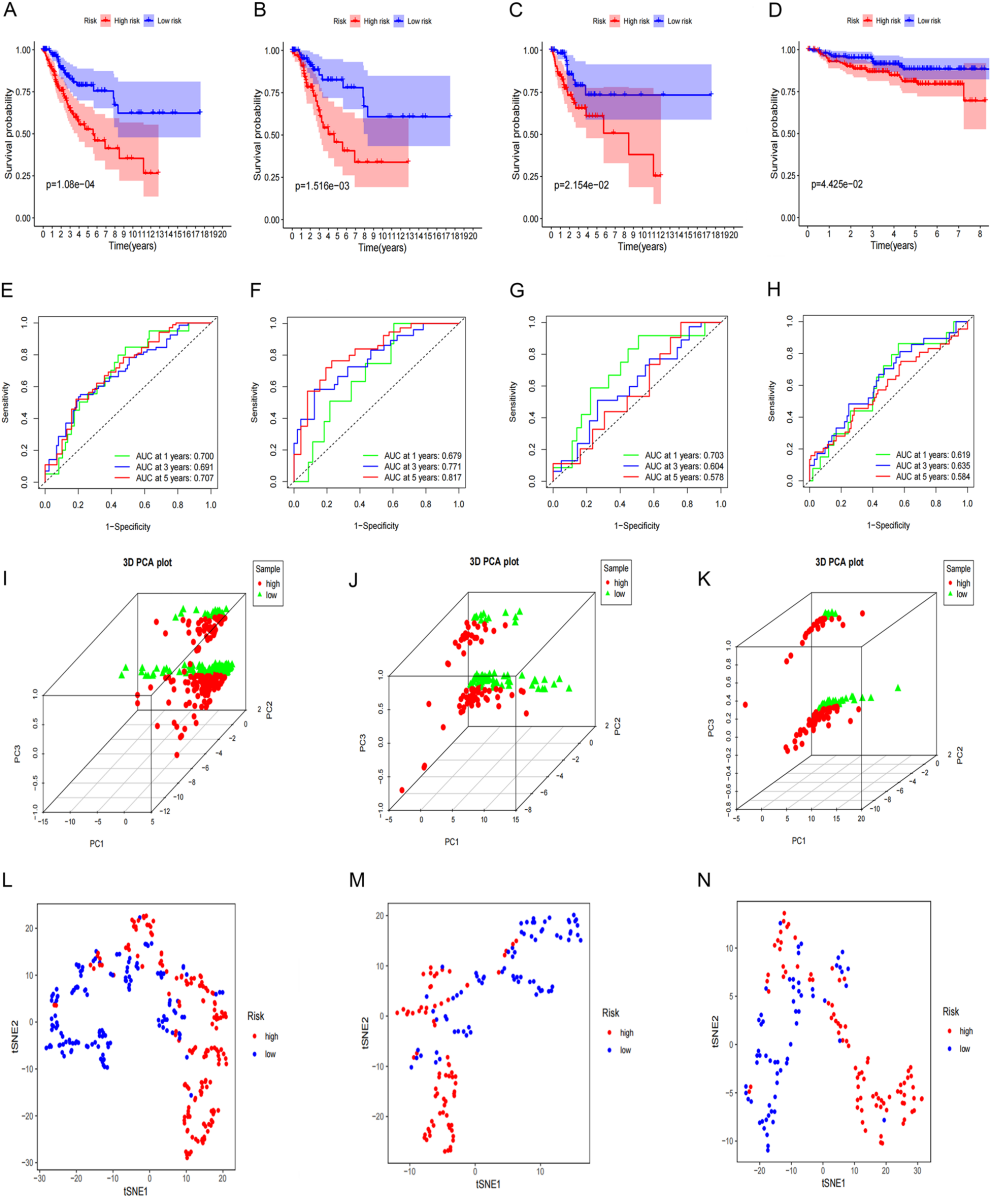
Relationship between the risk scores and clinicopathological factors. Kaplan–Meier curves were created to estimate OS for high- and low-risk groups from TCGA cohort (**A**), the two validation data sets (**B**–**C**) and GEO database (**D**); (**E**–**H**) TimeROC curves with two sets of validation datasets and GEO database; (**I**–**N**) Results of the principal components and t-SNE analysis in the TCGA cohort.

The risk scores were calculated for CESC patients in the TCGA cohort and the sample data were divided into low and high risk groups according to the median risk score. The distribution of risk scores and survival status and the validation datasets are shown in Fig. 6A–F. The Kaplan–Meier survival curve showed that CESC patients had lower survival rates and shorter survival time (Fig. 7A–C). Furthermore, the time-dependent ROC curve revealed that this model had good predictive power for overall survival of CESC patients. The area under the ROC curve (AUC) values were 0.700 (1 year), 0.691 (3 years) and 0.707 (5 years), respectively (Fig. 7E). The validation dataset confirmed the generalization ability of the model (Fig. 7F–G). To further validate the prognostic generality of the Cox proportional hazard model, we verified this prognostic model with a GEO testing cohort (GSE44001), which contains mRNA expression profiles and complete OS data from 300 CESC patients. The Kaplan–Meier survival curve showed that the survival of CESC-GEO cohort in the high-risk group was significantly lower than the low-risk group(Fig. 7D), and the AUC of the time-dependent ROC curve were 0.619 (1 year), 0.635 (3 years) and 0.584 (5 years) (Fig. 7H). All results revealed that consistent with other clinical parameters, the necroptosis-related mRNA risk score model has superior specificity and sensitivity. PCA and t-SNE analyses showed a significant difference in the distribution of CESC patients between the low-risk and CESC patients in the TCGA cohort (Fig. 7I–N).

### Independent prognostic value analysis of the risk score model

**Figure 8:**
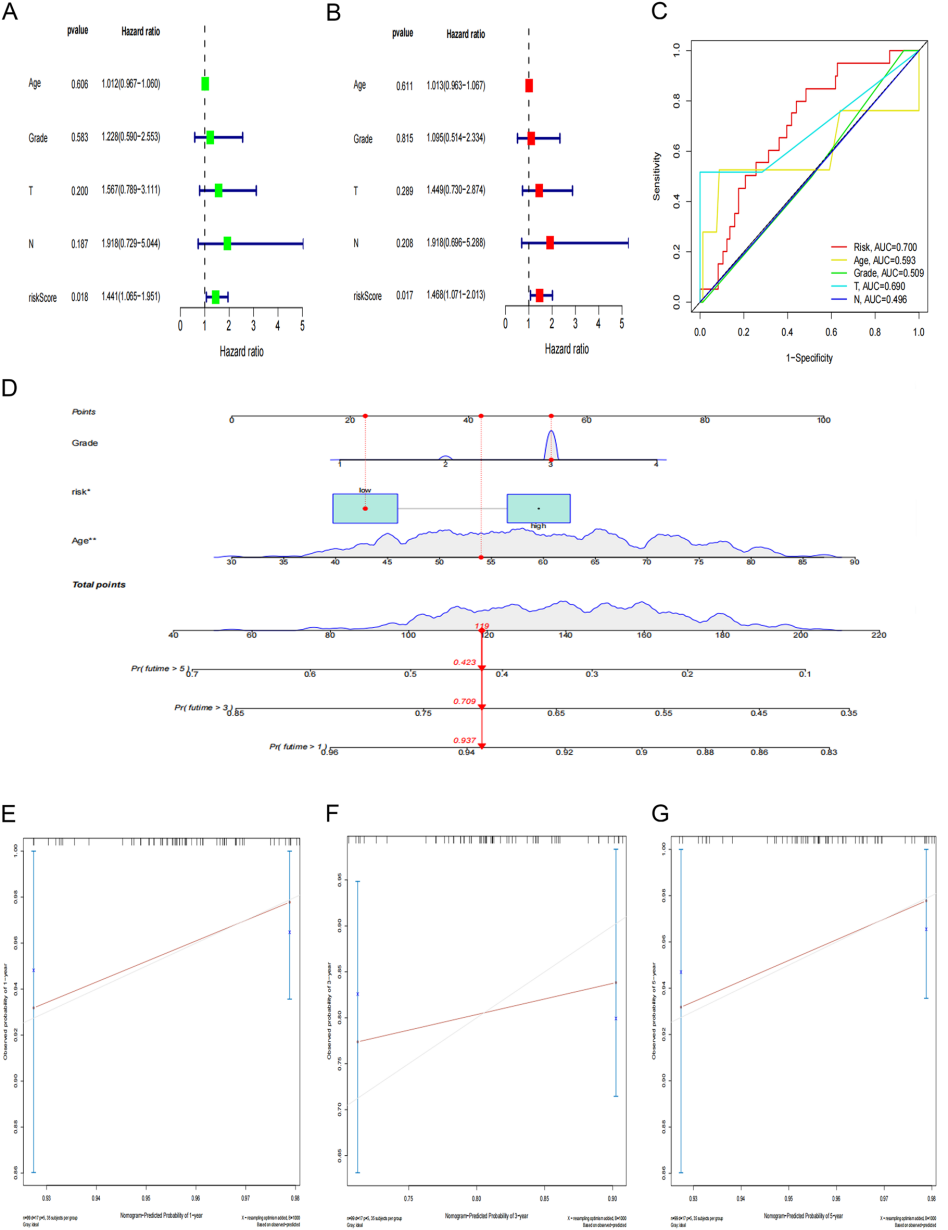
Independent prognostic value of risk score model. (**A**–**B**) Univariate and multivariate COX regression analysis on OS in the TCGA cohort. (**C**) Clinical ROC curves to forecast overall survival of patients. (**D**) Establishment of nomogram in forecasting OS rates at 1-year, 3-year and 5-year survival probabilities. (**E**–**G**) Nomogram calibration curves are used to investigate the predictive results and deviations of actual 1-year, 3-year and 5-year survival probabilities.

Univariate and multivariate COX regression analysis was used to evaluate whether this prognostic model could be an independent prognostic factor for CESC patients. In the TCGA cohort, the univariate COX regression analysis showed that the risk score was significantly correlated with overall survival in CESC patients [hazard ratio (HR) = 1.441; 95% confidence interval (CI) = 1.065–1,951; *P* < 0.05] (Fig. 8A). The multivariate COX regression further showed that the risk score was an independent prognostic factor for overall survival [HR = 1.468; 95%CI: 1.071–2.013; *P* < 0.001] (Fig. 8B). At the same time, the clinical ROC curves analysis showed that this risk signal had a higher predictive accuracy compared with the clinical data such as age, tumor grades and stages (Fig. 8C). The above results indicate that the prognostic model we constructed can serve as an independent prognostic factor.

Based on the results of the multivariate COX regression analysis, three variables were selected used to construct a nomogram model of the OS prognosis of CESC patients (Fig. 8D). All predictors were integrated with nomograms to predict the 1-year, 3-year and 5-year survival of MBC patients. The scores obtained from each variable were added together. The total score gained was able to predict the 1-year, 3-year and 5-year survival of MBC patients. The calibration curves were drawn according to the patient's 1-year, 3-year and 5-year survival, respectively. The calibration results used to evaluate the model (Fig. 8E–G) showed that all the calibration curves were in goodness of fit with the ideal curves, suggesting that the model had good accuracy and high predictive value in the prediction.

### Survival prediction of the risk score model was superior to traditional clinical indexes

**Figure 9:**
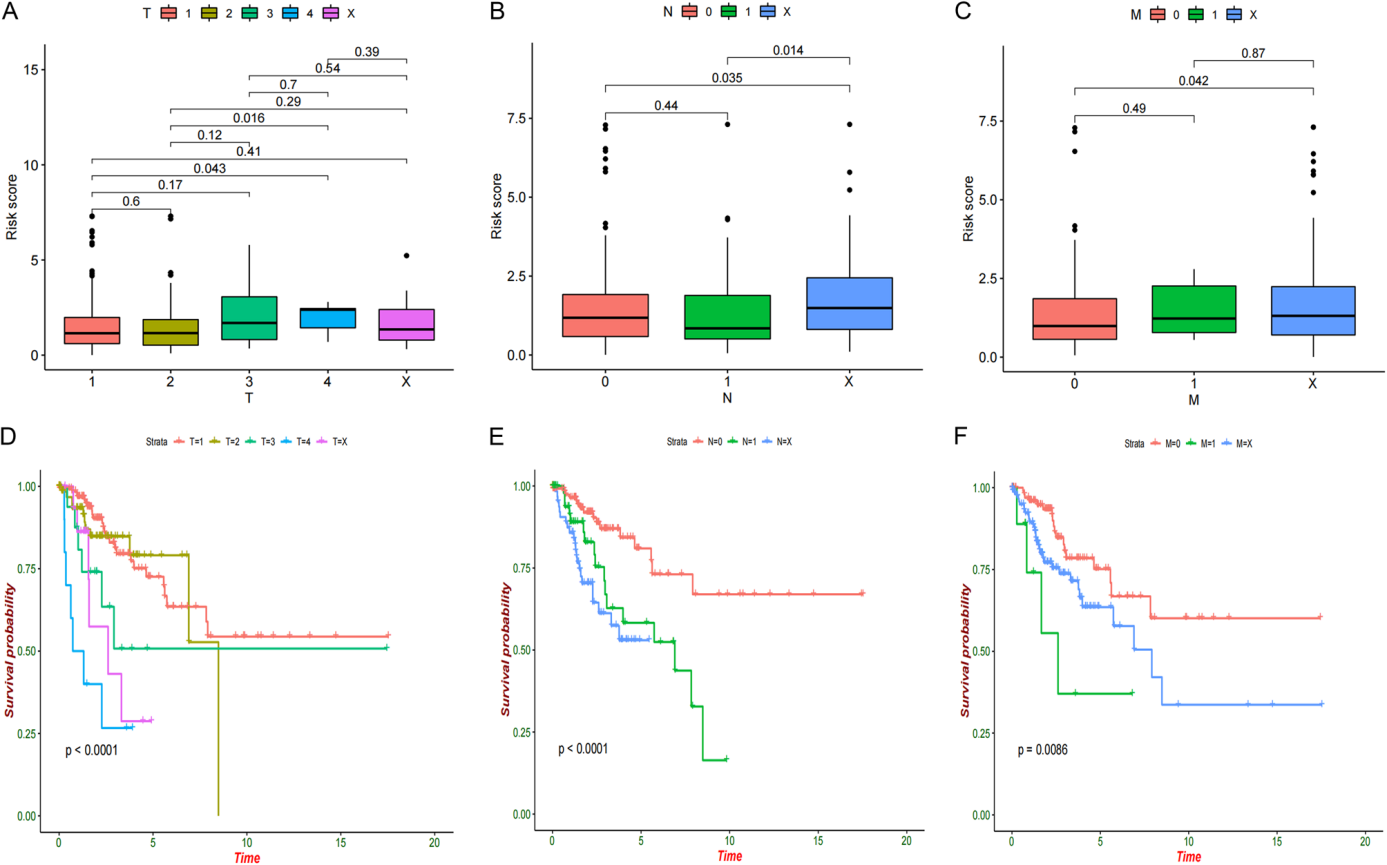
(**A**–**C**) Distribution of risk scores in TNMpathological stage. (**D**–**F**) The survival curve of TNM staging system.

The risk score was positively correlated with multiple clinicopathological factors in classic TNM staging system (Figs. 9A–C). The stratified survival analyses revealed that the OS of CESC patients was significantly different in different stages, and the patients had poor OS in T4 stage (Fig. 9D), N1 stage (Fig. 9E) and M1 stage (Fig. 9F). Moreover, the clinical ROC curves further showed that risk score model has superior specificity and accuracy in predicting survival relative to the classic TNM staging system (Fig. 8C). The combination of the prognostic model and the classic TNM staging system could improve the performance in predicting the survival of CESC patients.

### Functional enrichment analysis of differentially expressed genes in CESC patients

**Figure 10:**
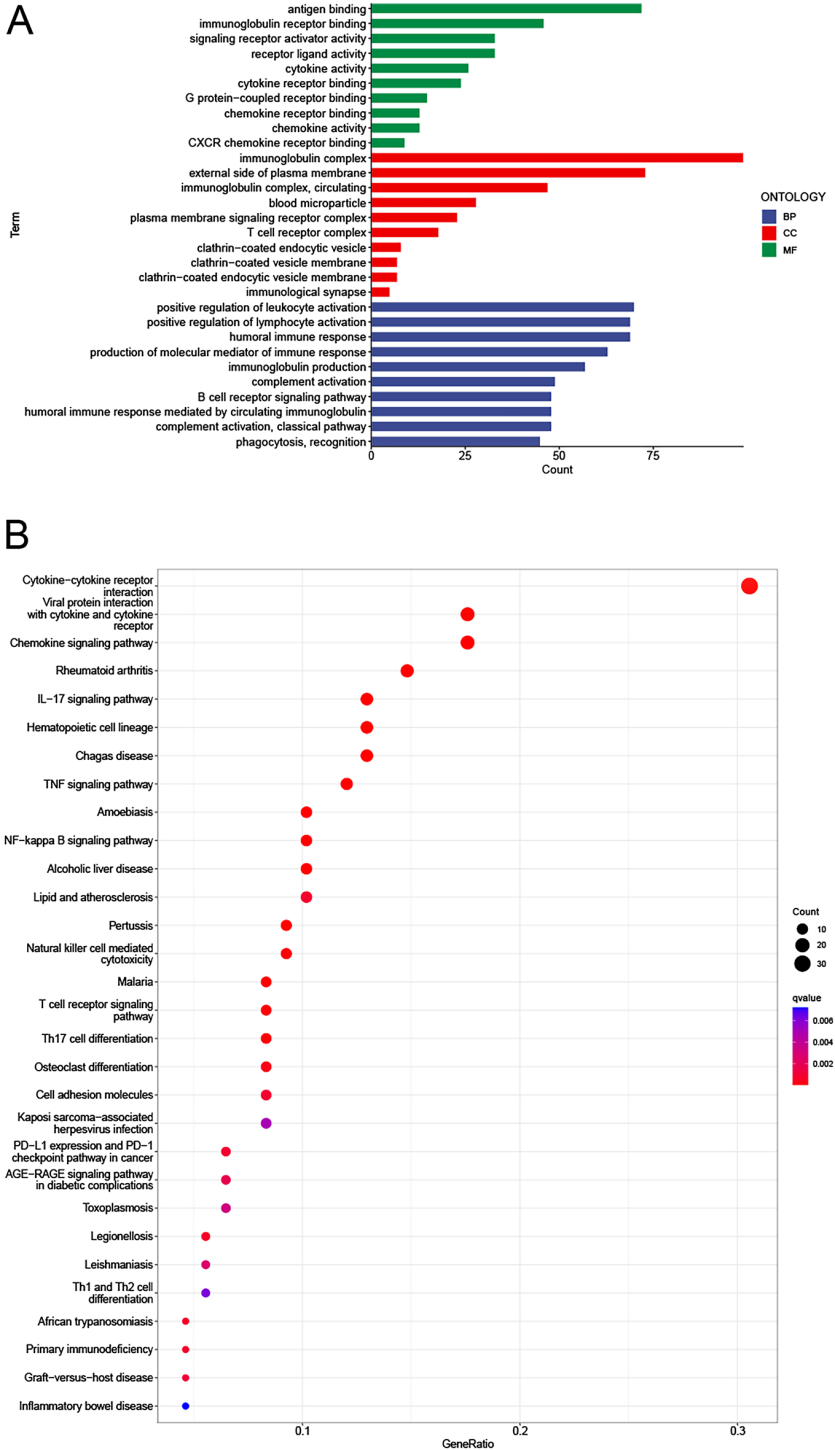
Functional enrichment analysis between the high and low risk groups of CESC patients. (**A**) Results of the GO enrichment analysis. (**B**) Results of the KEGG enrichment analysis.

In order to investigate the molecular heterogeneity and potential biological processes and pathways between the high and low risk groups, we identified 317 differentially expressed genes in the TCGA cohort [|log2 (fold change)|> 1, fdr < 0.05]. Using GO analysis, all differential genes were simultaneously enriched into the three biological relationships, namely BP, CC and MF. The results showed that the differential genes were mainly involved in antigen binding, immunoglobulin receptor binding and so on biological processes. Its products were mainly involved in cellular components including immunoglobulin complex, lateral plasma membrane and circulating immunoglobulin complex, etc., and played a role in biological molecular function, including positive regulation of leukocyte activation, positive regulation of lymphocyte activation and humoral immune response, etc. (Fig. 10A). The signaling pathways involved mainly included the cytokine-cytokine receptor interaction, the interaction of viral protein with cytokines and their receptors, and chemokine signaling pathways, etc. (Fig. 10B).

### Discovery of signaling pathways by GSEA

**Figure 11:**
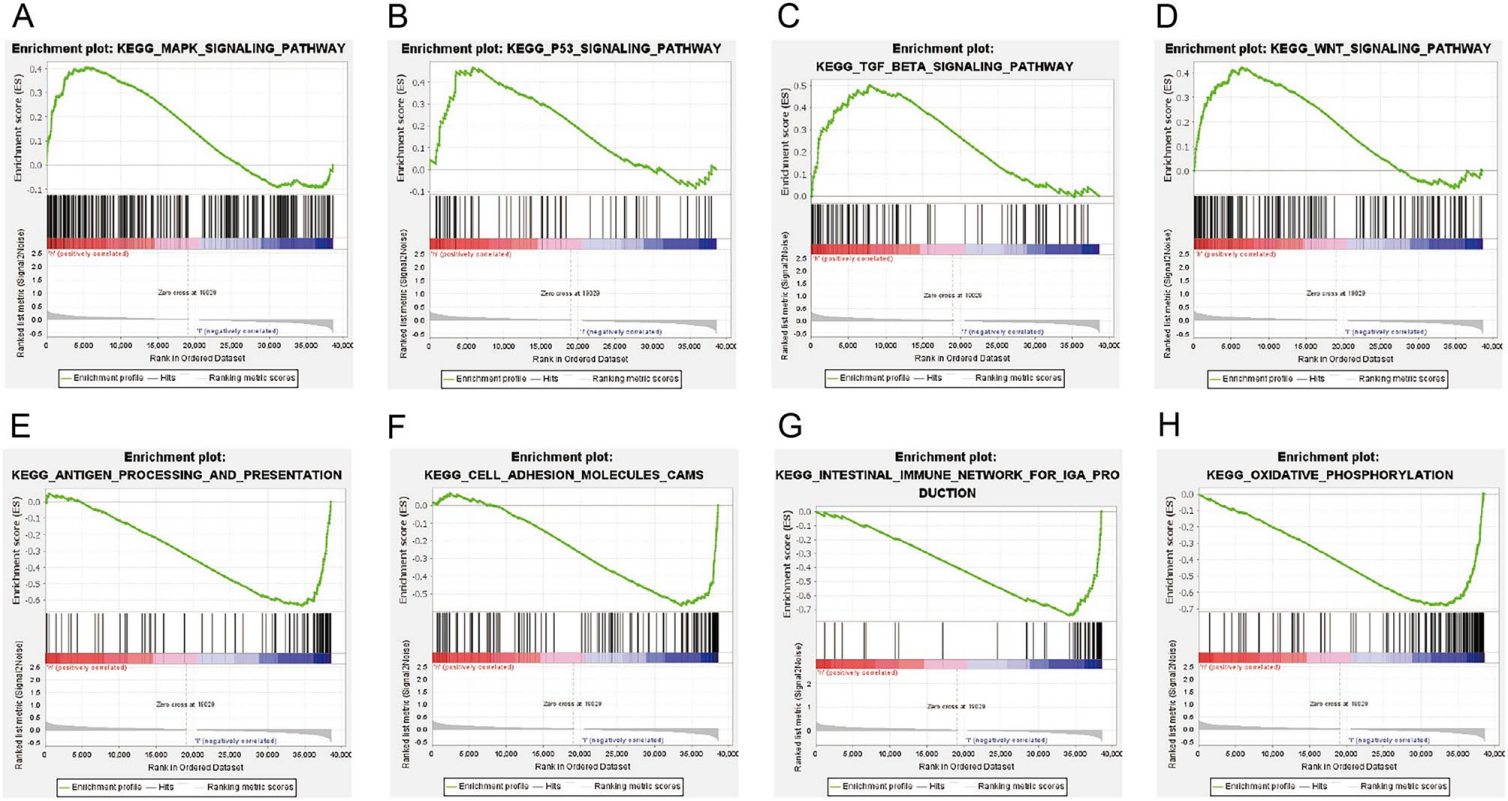
Enrichment plots from the gene set enrichment analysis. (**A**–**D**) Signaling pathways enriched in the high-risk CESC samples; (**E**–**H**) Biological processes enriched in the low-risk group of CESC patients.

Signaling pathways such as TGF-β, MAPK, Wnt and P53 were enriched in the NRmRNAs high-risk CESC phenotype (Fig. 11A–D). The antigen treatment and presentation, cell adhesion molecules (CAMS) and intestinal immune network produced by immunoglobulin and oxidative phosphorylation were enriched in the NRmRNAs low-risk CESC phenotype (Fig. 11E–H).

### Close correlation of the risk score model with immunity and M6A

**Figure 12:**
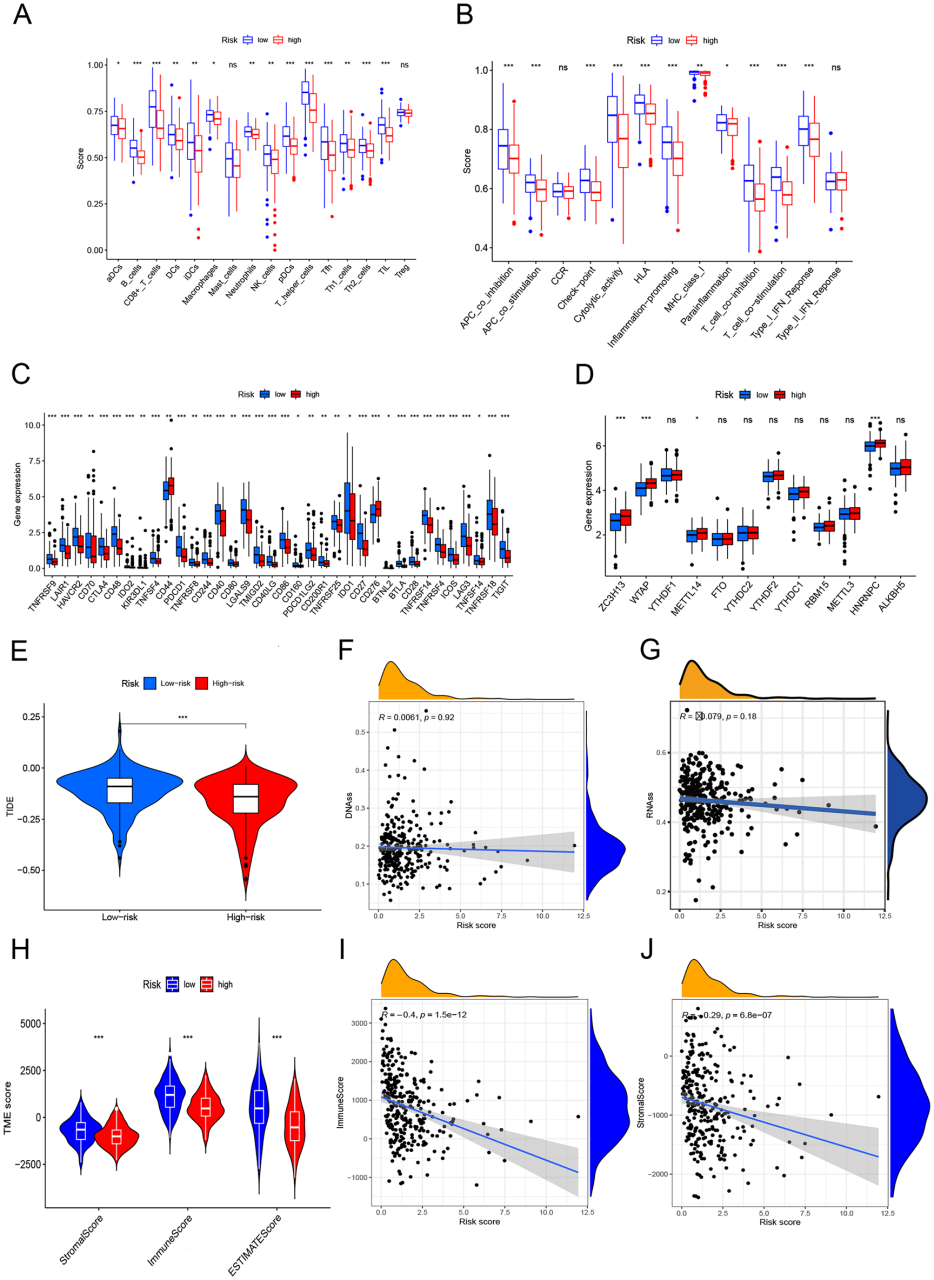
Immune correlation analysis. Boxplots of immune cell (**A**) and immune-related function (**B**) scores in the two risk subgroups. The differential expression based on the immune checkpoints (**C**), m6A-related genes (**D**) and TIDE scores (**E**) between the low-risk and high-risk groups. (**F**–**G**) Correlation between the risk scores and stem cells. (**H**) Violin plots show that the proportion of TIME scores vary between the high-risk and low-risk groups. (**I**–**J**) Correlation among the immune cells, stromal cell scores and risk scores.

In order to further investigate the relationship between the risk scores and immunity, we adopted ssGSEA to quantify the degree of infiltration of different immune cells. The results showed that the infiltration of CD8^+^ T cells, plasmacytoid dendritic cells (pDCs), helper T cells, Tfh, Th2 and TIL cells varied significantly in the TCGA cohort between the high and low-risk groups (*P* < 0.001) (Fig. 12A). The level of immune-related function was generally higher in the low-risk group than in the high-risk group (Fig. 12B). In terms of immune checkpoints, immune-related genes were significantly different between the two risk subgroups except for CD44 and CD276 (*P* < 0.05). Besides, the expression levels in the low-risk subgroup were higher than those in the high-risk subgroup (Fig. 12C). As shown in Fig. 12D, the expression levels of M6A-related genes ZC3H13, WTAP, METTL14 and HNRNPC were significantly higher in the high-risk subgroup than in the low-risk subgroup (*P* < 0.05). In addition, the TIDE scores of NRmRNAs were higher in the low-risk group and there was significant difference between the two different risk subgroups (*P* < 0.001) (Fig. 12E). However, the data showed no significant correlation between the risk scores and the stem cell indexes in CESC patients (Fig. 12F–G). The analysis of immune cell infiltration data in TIME revealed that there was significant difference in immune microenvironment scores including immune scores and matrix scores between the two risk subgroups (*P* < 0.001). Moreover, the level of immune cell infiltration in the low-risk subgroup was higher than that in the high-risk group (Fig. 12H). Spearman correlation test showed a negative correlation between the risk scores and immune cells, stromal cells. See Fig. 12I–J.

### Relationship of the OS in CESC patients among the TMB groups and two risk subgroups

**Figure 13:**
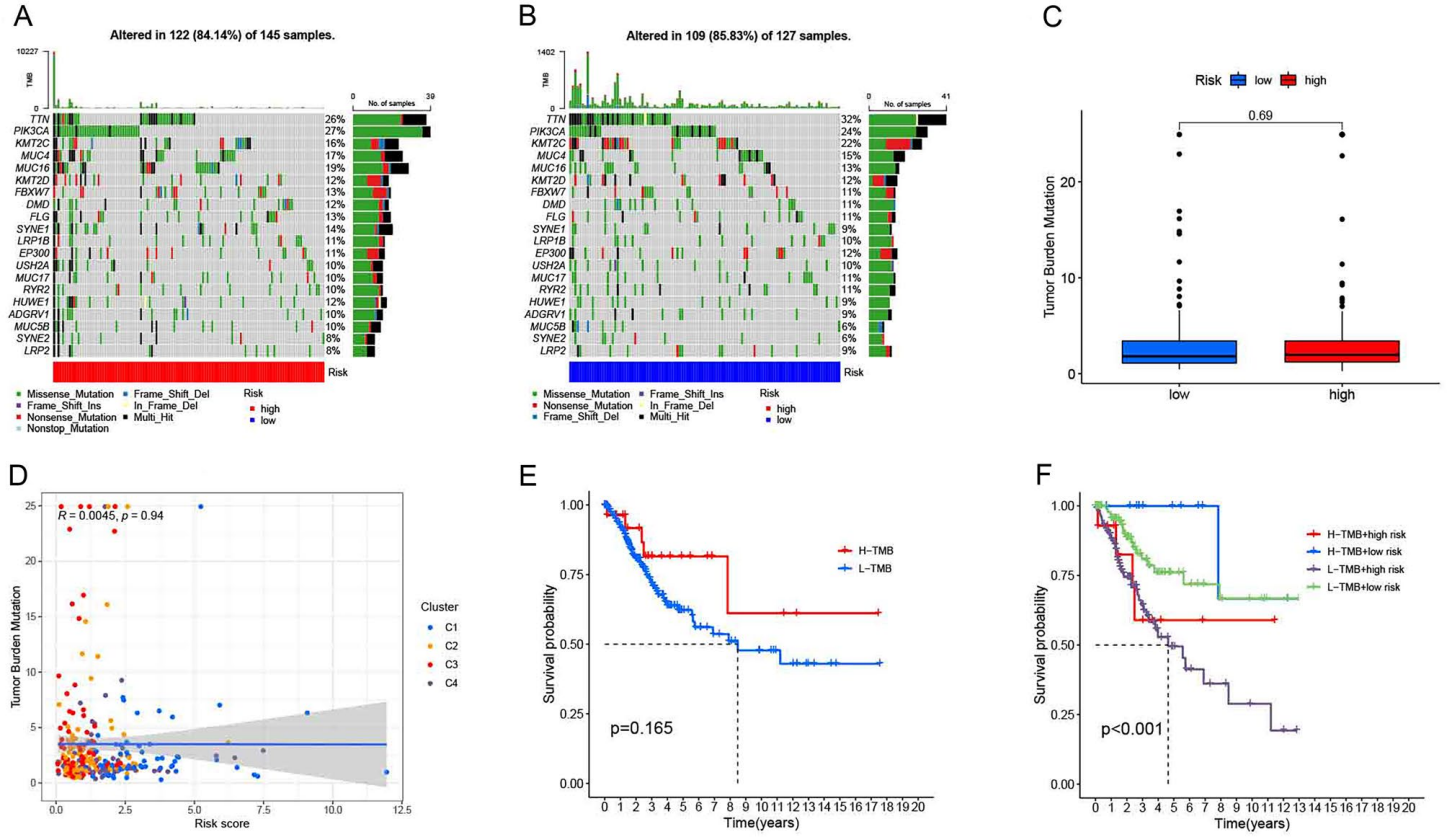
Correlation analysis among the TMB groups and two risk subgroups. (**A**–**B**) Distribution of the top 20 genes of TMB in the high and low risk groups; (**C**) Distribution of TMB in the high and low risk groups; (**D**) Correlation between the TMB and risk scores; (**E**–**F**) Relationship of overall survival in CESC patients among the TMB subgroups, high-risk and low-risk groups.

In the genetic mutation profile of the high-risk group, 122 (84.14%) out of 145 CESC patients had genetic mutations (Fig. 13A) and 109 (85.83%) out of 127 CESC patients had genetic mutations in the low-risk group (Fig. 13B). The difference in TMB was no significant in the TCGA cohort samples between the high and low risk groups (Fig. 13C). There was no significant correlation between the TMB levels and risk scores in the CESC patient samples (Fig. 13D). The TMB levels of CESC patients were calculated. A total of 288 CESC samples and combined TMB data and clinical information generated from 288 CESC samples were yielded by combining TMB data with clinical information (with 16 TMB and survival information samples removed). According to the best cutoff value (TMB = 1.894737) obtained based on X-Tile, all samples were divided into two groups, with 136 samples each in the high TMB and low TMB groups. Kaplan–Meier analysis showed that the CESC patients with high TMB levels had a high overall survival (*P* < 0.01). See Fig. 13E–F.

### Analysis of differences in drug sensitivity between the high and low risk groups

**Figure 14:**
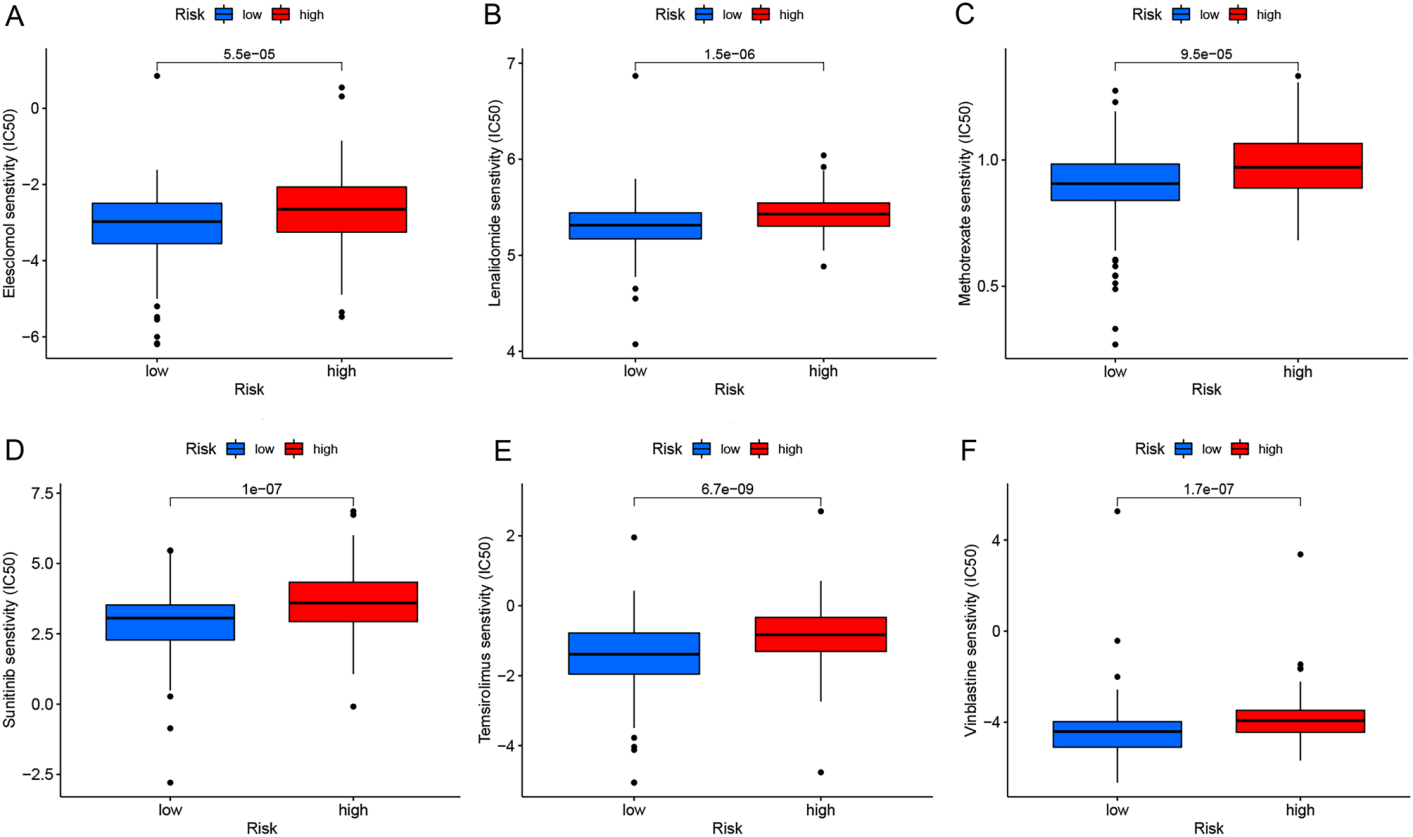
(**A**–**F**) Distribution of drug sensitivity between the two-risk subgroups.

Drug sensitivity analysis showed that there was a difference in drug sensitivity between the high and low risk groups (*P* < 0.01). Moreover, NRmRNAs risk scores in CESC patients were negatively correlated with the sensitivity of most drugs (Fig. 14A–F). Therefore, drugs targeting NRmRNAs may play a therapeutic role in CESC patients in the low-risk group and work better than patients in the high-risk group.

## Discussion

Cervical carcinoma (CC) is the fourth largest female cancer worldwide and it is one of the leading causes of cancer deaths among women^21^. The methods to manage the CC mainly include pre-onset prevention and post-onset treatment. The prevention of the CC includes early screening and having a shot of cervical cancer vaccine^22^. The surgery is mainly applied in the treatment of the CC, plus a comprehensive treatment regimen^23^ of cisplatin-based chemotherapy in combination with adjuvant brachytherapy. Although the diagnosis and treatment strategies of CC have been improved, the CC has a high degree of malignancy and a strong ability of metastasis and invasion. It often has no obvious symptoms and signs in the early stage. Therefore, it is easy to miss diagnosis or have misdiagnosis. The fatality rate is rather high^24^. Therefore, analyzing the gene expression profiles and clinicopathological characteristics of CC patients and exploring potential prognostic biomarkers are important for the early prevention, treatment of disease and the improvement of patient survival.

With the rapid development of high-throughput sequencing and gene microarrays, the relationship between some gene alterations and diseases can be detected, thus providing some theoretical evidence for the diagnosis and prognosis of diseases. Previous studies have shown that CASP8 may play an oncogene role^25^ in glioma. It may also be used to predict the survival of patients with hepatocellular carcinoma (HCC) and the response to the immunotherapy^26^. Having combined with the somatic mutation data in this study, it can be speculated that the higher mutation frequency of CASP8 may be significantly associated with the poor prognosis of the patients. Interleukin-12 (IL-12) is a proinflammatory cytokine of heterodimeric structure. IL12A, a gene that encodes P35 is localized within 3p12-3q13.2^27^. At present, IL-12 has been confirmed to have significant antitumor activity in lung cancer^28^ and gastric cancer^29,30^, etc. It is one of the potential effective cytokines in the antitumor immunotherapy. Its tumor suppressor mechanism mainly depends on its capacity^27^ to activate the Th1-acquired immunity and CTL, thus immunoregulating the tumor microenvironment, inhibiting the generation of surrounding tumor blood vessels, and then reducing the nutrient supply^31^ to tumor cells. Gene copy number variation is a common form of genetic multisite mutations that may affect the biological phenotype and heterogeneity of tumors, as well as drive the complex growth of tumors. It is generally believed that the amplified sections in the tumor genome generally contain important oncogenes, while the missing sections often contain the key anti-oncogenes^32^. The presence of significant copy number amplification of IL12A in this study suggests that the IL12A may act as an oncogene in CESC. This finding may be used in early CESC cancer diagnosis and cancer prevention program in the near future. However, further clinical research is required for the specific value.

In this study, we have first introduced the consensus clustering analysis to divide NRmRNAs into four different subgroups and systematically investigated the correlation among the OS, PFS, TME, immune cell infiltration with NRmRNAs. TME is a complex local tissue environment where tumor cells are settled, including various cell types (endothelial cells, fibroblasts, immune cells, neuroendocrine cells, adipocytes and mesenchymal cells, etc.) surrounding the tumor cells as well as extracellular components (cytokines, growth factors, hormones and extracellular matrix, etc.)^33^. TME not only plays a key regulatory role^34^ in tumor progression and metastasis, but also mediates tumor drug resistance^35^ through multiple mechanisms. The TME is infiltrated with various immune cell subsets, including effector and inhibitory immune cells, which play an important role in predicting tumor prognosis and the treatment tolerance through direct contact or interaction between the chemokines and tumor cells. Among them, CD8^+^ T cells have the tumor killing function while regulatory T cells (regulatory T, Tregs) attenuate the effector T cell activity and promote the immunosuppression of TME. The prognostic value^36^ is evaluated by comprehensive determination of the proportion of inhibitory cells such as intratumoral CD8^+^ T cells and Tregs. The infiltration of CD8^+^ T cells in TME could be a biomarker for predicting the efficacy of anti-PD-1/PD-L1 therapy. Moreover, it is found that IL-2 and others can effectively enhance T cell-mediated tumor immunotherapy effect by inhibiting Tregs function, targeting exhausting Tregs, or interfering with their recruitment to TME. T cell-infiltrating TME may produce the optimal response^37,38^ to therapies that inhibit the immune system. In this paper, Tregs from Cluster2 and CD8^+^ T cells from Cluster4 are more than those in the other two groups. Moreover, the difference in ESTIMATEScore, ImmuneScore and TumorPurity is statistically significant between the two groups (p < 0.1). These results indicate that Tregs and CD8^+^ T cells have the potential to be candidate predictive biomarkers of immunotherapy response in patients of Cluster2 and Cluster4 groups. The individualized treatment strategies will be optimized for the patients in the different CESC subtypes by using precisely targeted TME immunotherapy or drug combination.

Furthermore, we have constructed the first risk score model containing three NRmRNAs (CXCL8, CLEC9A and TAB2) in the TCGA cohort with independent prognostic ability in CESC patients, as well as validated the dataset established by random allocation. Its performance and stability were further validated in another testing dataset (GSE44001) from GEO database. Interleukin 8 (CXCL8/IL8) is a chemokine secreted by activated tumor cells, which regulates the proliferation and self-renewal^39^ of inflammatory factors and tumor stem cells (CSCs) by acting on CXCR1/2 in the tumor microenvironment. Studies have shown that CXCL8, as an inflammatory marker, is identified as a prognostic marker^40^ for oral cancer. The CXCL8 plays an important role^41^ in proliferation and metastasis by inducing angiogenic factors in HCC cells such as VEGFA. Meanwhile, the CXCL8 receptor (CXCR1 and CXCR2) antagonist^42^ may be a potential targeted therapeutic method for HCC. The CXCL8 inhibits the expression of ER^+^ in endometrial cancer (EC) cells, which may be closely related^43^ to clinical stages and tumor invasion. Type lectin domain containing 9A (CLEC9A) also known as DC-NK lectin group receptor-1 (DNGR-1), is a surface molecule of dentritic cells (DC) found in 2008. It is a specific receptor derived from members of the type 2 transmembrane C-type lectin domain family^44–46^. DC is a dedicated presenting cell that connects innate and adaptive immunity in vivo. CLEC9A targeted antigen to DC may stimulate the body to produce CTL responses, identify tumor cell surface-related tumor antigens, and promote the death^47,48^ of tumor cells through cytotoxicity. Sancho et al.^49^ have constructed a model of mouse melanoma and inoculated the melanoma-derived epitope-coupled anti-CLEC9A monoclonal antibodies into mice before tumorigenesis (prevention model) and after development (treatment model). The results show that targeting CLEC9A may produce a strong long-term CTL response with a significant decrease in the number of tumor cells and play an important role in inducing anti-tumor immune response and preventing tumors in the body. In addition, some studies have shown that the abnormal CLEC9A gene expression after radiotherapy of nasopharyngeal carcinoma (NPC) is closely related to the prognosis of head and neck squamous cell carcinoma (HNSCC). The interference of CLEC9A may play a key role^50^ in the regulation of radiotherapy response. Previous studies have found that the transforming growth factor β-activated kinase 1 binding protein 2 (TAB2) gene is a key regulatory factor^51^ of the NF-κB signaling pathway. The knockdown of the TAB2 gene significantly inhibits cell proliferation and induces cell apoptosis. Moreover, the regulatory mechanism of TAB2 in cancer stem cells (CSCs) is related to promoting the malignant transformation and invasiveness of CC, which is a prognostic marker of CC^52^. Our results are consistent with these studies. The multi-omics data confirm that our risk score is a specific and sensitive indicator for predicting OS in patients with CES. We report the clinical significance of NRmRNAs for the prognosis and immune response in CESC patients for the first time. Screening for accurate and effective prognostic markers is important for guiding clinical personalized therapy and the development of precision medicine.

In order to investigate the mechanisms underlying the occurrence and development of CESC, a detailed biological annotation and description of gene product function have been performed using gene function analysis and pathway analysis. The results of GO enrichment analysis suggest that the differentially expressed genes are mostly enriched in immune-related biological processes such as immunoglobulin receptor binding. Their products are mainly involved in immunoglobulin complexes and have biological molecular functions such as humoral immune response. Meanwhile, KEGG analysis reveals that differentially expressed genes are enriched in anticancer immunomodulatory pathways, including natural killer cell mediated cytotoxicity, NF − kappa B signaling pathway and T cell receptor signaling pathway, etc. The above-mentioned results suggest that the occurrence and development of CESC may be closely related to immunity. Furthermore, this study has predicted the role of NRmRNAs in CESC using GSEA analysis. The results show that the CESC high-risk phenotypes are significantly enriched in the signaling pathways such as TGF-, MAPK and Wnt. Transforming growth factor-β (TGF-β) has a bidirectional regulation effect in tumor tissues at different stages, showing low expression in cervical tissues of patients with phase I-III cervical intraepithelial neoplasia and high expression^53^ in cervical cancer tissues. In vitro animal data suggest that the autophagy of TGF-β signaling pathway may be involved in the occurrence^54^ of cervical cancer. Furthermore, TGF-β may affect the occurrence and development^55,56^ of HPV infection-related cervical carcinoma by interacting with key pathogenic proteins in HPV infection. Mitogen-activated protein kinase (MAPK) is a kind of oncogenic signaling pathway^57^. Strongly phosphorylated P38/MAPK is an independent risk factor associated with poor prognosis due to cancer. Activation of P38 MAPK leads to the activation of Nuclear factor erythroid 2-related factor 2 (Nrf2), thereby leading to the acquired drug resistance^58^ to temozolomide in glioma cells. Wnt/β-catenin pathway promotes the proliferation and invasion^61^ of ovarian cancer by participating in ovarian tumor angiogenesis^59^ and immune escape^60^. These findings suggest that NRmRNAs may have a potential role in regulating CESC progression. Its signaling pathways involved in this process may lead to malignant phenotypes such as proliferation, invasion and metastasis of cancer cells and poor prognosis such as drug resistance.

For many years, the patients with recurrent and advanced metastatic CC who cannot be treated surgically have many problems^62^ such as poor prognosis, significantly declined quality of survival and high early mortality, etc. On account of this, with the deepening of tumor immunity research and the continuous maturity of new gene editing techniques, immuno-oncology has become a potential new strategy to improve the prognosis of cervical cancer patients. Clinical studies have found that the immunotherapy is superior to the traditional anti-tumor therapies. Strategies including immune checkpoint inhibitors and adoptive cell therapy (ACT) developed based on T cells have generated positive objective response rates in patients who do not respond to conventional therapies. As shown in the ssGSEA results, there is a significant difference in proportion of tumor-infiltrating immune cells between the high and low risk groups. Besides, the tumor-killing immune cells^63,64^ such as CD8^+^ T cells, Macrophages and NK cells infiltrating in CESC tissue are significantly reduced in the high-risk group compared to the low-risk group. Thus it can be seen the necroptosis is significantly correlated with the proportion of tumor-infiltrating immune cells in CESC. Moreover, most CESC patients in the low-risk subgroup have high molecular expression levels of immune checkpoints, indicating that it is more likely to benefit from immune checkpoint inhibitors. NRmRNAs may be helpful in predicting the efficacy of immune checkpoint blockade (ICB) therapy in CESC patients. Regarding TIME, the level of immune cell infiltration is significantly higher in the low-risk subgroup than in the high-risk group. Moreover, the Spearman correlation test reveals that the risk score is negatively correlated with immune cells and stromal cells. Therefore, immunotherapy may have a higher response rate and better efficacy in patients of the low-risk subgroup. TMB has been confirmed as a biomarker to predict ICB efficacy in breast cancer and non-small cell lung cancer. Its expression level is positively correlated^65,66^ with the survival time of tumor patients. Our study findings similarly confirm this view, suggesting that TMB may play an important role in predicting the efficacy and prognosis of immunotherapy in CESC patients. Previous literature show that m6A expression level in cervical cancer tissues is closely related^67^ to tumor typing, staging, progression, metastasis and recurrence. Our study has found that there is a significant difference in the expression levels of M6A-related genes ZC3H13, WTAP, METTL14, and HNRNPC between the high and low risk subgroups, which may provide new directions and ideas for the treatment of CESC.

## Conclusion

In conclusion, this study has introduced RNA sequencing data from TCGA to construct a Cox proportional hazards model consisting of three necroptosis-related mRNA as a biomarker to identify prognostic and immune responses, so as to provide reference for further investigation of the pathogenesis and drug treatment of CESC. However, there are some limitations in this study. Firstly, we have constructed the model using the TCGA dataset and validated the generalization ability of the model using the random allocation dataset. Nevertheless, more independent external datasets and further in vivo and in vitro experimental studies are still needed to validate the model. In the future, we will verify the accuracy of the results of this study at the molecular, cellular and tissue levels. Further screening of accurate and effective prognostic markers, the design of multiple combined targeted therapeutic strategies and the exploration of new therapeutic targets are bound to be the focus of future research. The detection and targeted therapy acting on the early diagnosis and prognosis of CESC may be a powerful supplement to the existing clinical means, which will help to reduce the pain and economic burden of patients.

## Data Availability

The datasets generated and/or analysed during the current study are available in the [The Cancer Genome Atlas (TCGA)] repository, [https://portal.gdc.cancer.gov/] and UCSC Xena repository [https://xenabrowser.net/datapages/]. Details of R software: R is a free software environment for statistical computing and graphics. It compiles and runs on a wide variety of UNIX platforms, Windows and MacOS. R version 4.1.2; link: https://www.r-project.org/.
